# Image-Derived Input Function Derived from a Supervised Clustering Algorithm: Methodology and Validation in a Clinical Protocol Using [^11^C](*R*)-Rolipram

**DOI:** 10.1371/journal.pone.0089101

**Published:** 2014-02-20

**Authors:** Chul Hyoung Lyoo, Paolo Zanotti-Fregonara, Sami S. Zoghbi, Jeih-San Liow, Rong Xu, Victor W. Pike, Carlos A. Zarate, Masahiro Fujita, Robert B. Innis

**Affiliations:** 1 Department of Neurology, Gangnam Severance Hospital, Yonsei University College of Medicine, Seoul, South Korea; 2 Molecular Imaging Branch, National Institute of Mental Health, National Institutes of Health, Bethesda, Maryland, United States of America; 3 University of Bordeaux, CNRS, INCIA, UMR 5287, Talence, France; 4 Experimental Therapeutics and Pathophysiology Branch, National Institute of Mental Health, National Institutes of Health, Bethesda, Maryland, United States of America; University of Manchester, United Kingdom

## Abstract

Image-derived input function (IDIF) obtained by manually drawing carotid arteries (manual-IDIF) can be reliably used in [^11^C](*R*)-rolipram positron emission tomography (PET) scans. However, manual-IDIF is time consuming and subject to inter- and intra-operator variability. To overcome this limitation, we developed a fully automated technique for deriving IDIF with a supervised clustering algorithm (SVCA). To validate this technique, 25 healthy controls and 26 patients with moderate to severe major depressive disorder (MDD) underwent T1-weighted brain magnetic resonance imaging (MRI) and a 90-minute [^11^C](*R*)-rolipram PET scan. For each subject, metabolite-corrected input function was measured from the radial artery. SVCA templates were obtained from 10 additional healthy subjects who underwent the same MRI and PET procedures. Cluster-IDIF was obtained as follows: 1) template mask images were created for carotid and surrounding tissue; 2) parametric image of weights for blood were created using SVCA; 3) mask images to the individual PET image were inversely normalized; 4) carotid and surrounding tissue time activity curves (TACs) were obtained from weighted and unweighted averages of each voxel activity in each mask, respectively; 5) partial volume effects and radiometabolites were corrected using individual arterial data at four points. Logan-distribution volume (*V*_T_/*f*_P_) values obtained by cluster-IDIF were similar to reference results obtained using arterial data, as well as those obtained using manual-IDIF; 39 of 51 subjects had a *V*_T_/*f*_P_ error of <5%, and only one had error >10%. With automatic voxel selection, cluster-IDIF curves were less noisy than manual-IDIF and free of operator-related variability. Cluster-IDIF showed widespread decrease of about 20% [^11^C](*R*)-rolipram binding in the MDD group. Taken together, the results suggest that cluster-IDIF is a good alternative to full arterial input function for estimating Logan-*V*_T_/*f*_P_ in [^11^C](*R*)-rolipram PET clinical scans. This technique enables fully automated extraction of IDIF and can be applied to other radiotracers with similar kinetics.

## Introduction

[^11^C](*R*)-rolipram is a positron emission tomography (PET) radioligand for phosphodiesterase 4 (PDE4), an enzyme that metabolizes cyclic adenosine monophosphate (cAMP). By imaging the cAMP cascade, [^11^C](*R*)-rolipram may offer unique insights into the internal machinery of brain cells. Our laboratory previously showed that patients with major depressive disorder (MDD) have widespread and significantly reduced [^11^C](*R*)-rolipram binding compared to healthy subjects [1].

[^11^C](*R*)-rolipram can be reliably quantified by a two-tissue compartment model and a 90-minute brain scan [2]. Alternative techniques for [^11^C](*R*)-rolipram quantification, both at the region and voxel level, include the Logan plot, the hierarchical basis function method, and spectral analysis [3]. Because of widespread distribution of PDE4 in whole gray matter and no region for reference tissue, all of these techniques require measuring arterial input function, which is a labor-intensive and invasive procedure. We previously found that the workload for obtaining the input function could be considerably reduced by using an image-derived input function (IDIF) from carotid arteries [4]. In that study, IDIF curves were derived from manual segmentation of the carotid and background regions; however, manual segmentation is a time-consuming (and tedious) task that suffers from intra- and inter-operator variability.

Several algorithms have been proposed in the literature for more objective segmentation of PET images. Some are semi-automated, meaning that they still require intervention from the operator [5], [6]. Nevertheless, many completely automatic algorithms based on the unsupervised clustering of voxels are now available [7]–[11]. However, unsupervised clustering algorithms define tissue classes in a data-dependent manner that may not accurately describe the underlying physiology and, therefore, introduce instability or inaccuracies in the grouping [12]. When the pharmacokinetic behavior of the different tissues is already known, segmentation can be achieved in a simple and robust way by taking advantage of predefined kinetic classes [13]. Indeed, Turkheimer and colleagues [12] developed a supervised clustering algorithm (SVCA) for the automatic extraction of reference tissue curves. This algorithm was validated in [^11^C](*R*)-PK11195 scans, and the reference time-activity curve (TAC) was extracted by discriminating regions with and without pathologically increased [^11^C](*R*)-PK11195 binding.

In the current study, we examined a new objective method to automatically segment PET images for IDIF by applying SVCA. In addition to extracting reference tissue curves, the SVCA can also be used to extract the blood signal from the intracranial vessels in a fast, objective, and reproducible way. After opportune partial volume effect and spill-in correction, this signal could be used as an IDIF for kinetic modeling. The present study sought to: 1) adapt Turkheimer’s SVCA to our PET data in order to automatically derive an IDIF–without any intervention from the operator–for calculating [^11^C](*R*)-rolipram binding; and 2) replicate the findings of a previous clinical study that compared [^11^C](*R*)-rolipram binding between healthy subjects and MDD patients using cluster-IDIFs [1].

## Methods

### Radioligand Preparation

[^11^C](*R*)-rolipram was synthesized as previously described [14] and according to our Investigational New Drug Application #73,149, submitted to the US Food and Drug Administration. A copy of our application is available at: http://pdsp.med.unc.edu/snidd/nidpulldownPC.php. The radioligand was obtained in high radiochemical purity (>99%).

### Subjects

Study subjects included 25 healthy individuals (9 females, 16 males, 37±11 years old, body weight = 83±16 kg) and 28 patients with moderate-to-severe MDD (9 females and 19 males, 36±11 years old, body weight = 86±23 kg) [1]. Two of the MDD subjects were excluded because their arterial input function was obtained only for the first 60 minutes. See [1] for inclusion criteria and clinical characteristics of the subjects. This study was approved by the Combined Neuroscience Institutional Review Board of the NIH Intramural Research Program, and all subjects provided written informed consent to participate.

### Measurement of [^11^C](*R*)-rolipram in Plasma

All blood samples (1 mL each) were drawn from the radial artery at 15-second intervals for the first 150 seconds, followed by 3 mL samples at 3, 4, 6, 8, 10, 15, 20, 30, 40, and 50 minutes, and 4.5 mL at 60, 75, and 90 minutes. The plasma TAC was corrected for the fraction of unchanged radioligand [14], [15]. The plasma free fraction (*f*_P_) of [^11^C](*R*)-rolipram was determined by ultrafiltration [16]. Assay-by-assay fluctuations in *f*_P_ measurement were corrected based upon *f*_P_ of a standard plasma sample assayed with the subject’s sample [17].

### PET Scans

PET images were acquired with an Advance scanner (GE Healthcare, Milwaukee, WI). A head holder was used to minimize head movements during the scan. An 8-minute ^68^Ge transmission scan was obtained before injection of the radiotracer for attenuation correction. The mean injected activity was 726±97 MBq for healthy subjects and 703±108 MBq for MDD patients. The emission scan was conducted for 90 minutes in 3D mode using frames of increasing length from 30 seconds to 5 minutes. After attenuation and a model-based scatter correction, PET images were reconstructed with filtered back projection and a Hanning filter in 128×128×35 matrix with 2×2×4.25 mm voxel size, resulting in a 7 mm image spatial resolution.

### Magnetic Resonance Image (MRI) Scans

T1-weighted structural MRIs were also obtained using either a 3-T Signa scanner (GE, Milwaukee, WI) or an Achieva 3-T MRI scanner (Philips Health Care, Andover, MA). We used a TFE (turbo field echo) sequence (TR = 8.1 msec, TE = 3.7 msec, flip angle = 8, matrix = 181×256×256, voxel size = 1×0.983×0.983 mm).

### Image Analysis

The merged automated anatomical labeling (AAL) template was used to measure regional TAC [18]. Predefined volumes of interest (VOIs) were positioned on the PET images using the following methods. The MR image from each subject was coregistered using SPM8 (Wellcome Department of Imaging Neuroscience; University College London, UK) to the average PET image created by summing all of the dynamic image frames. The MR and the realigned PET images were normalized to a standardized spatial array (Montreal Neurological Institute (MNI) space) based on transformation parameters from the MR images. The final VOIs included the following regions: thalamus (12.6 cm^3^), caudate (5.6 cm^3^), putamen (6.5 cm^3^), cerebellum (51.2 cm^3^), frontal (27.2 cm^3^), parietal (26.6 cm^3^), lateral temporal (25.0 cm^3^), occipital (31.2 cm^3^), anterior cingulate (7.5 cm^3^), and medial temporal (14.3 cm^3^) cortices.

### Creation of the IDIF

Three steps were involved in creating the IDIF: 1) the template was defined; 2) the whole blood curve was extracted from dynamic PET; and 3) partial volume effect and radiometabolites were corrected.

#### 1) Defining the template

To create the templates necessary for the clustering procedure, [^11^C](*R*)-rolipram images were used from 10 healthy subjects (8 males and 2 females, 743±7.0 MBq, 84.2±21.1 kg, 31.4±10.7 years old) who did not belong to the population described above. Scans for these subjects were acquired using the same machine and acquisition protocol as for the 51 subjects in the clinical study.

#### Creation of a ligand-specific PET template

**Figure 1:**
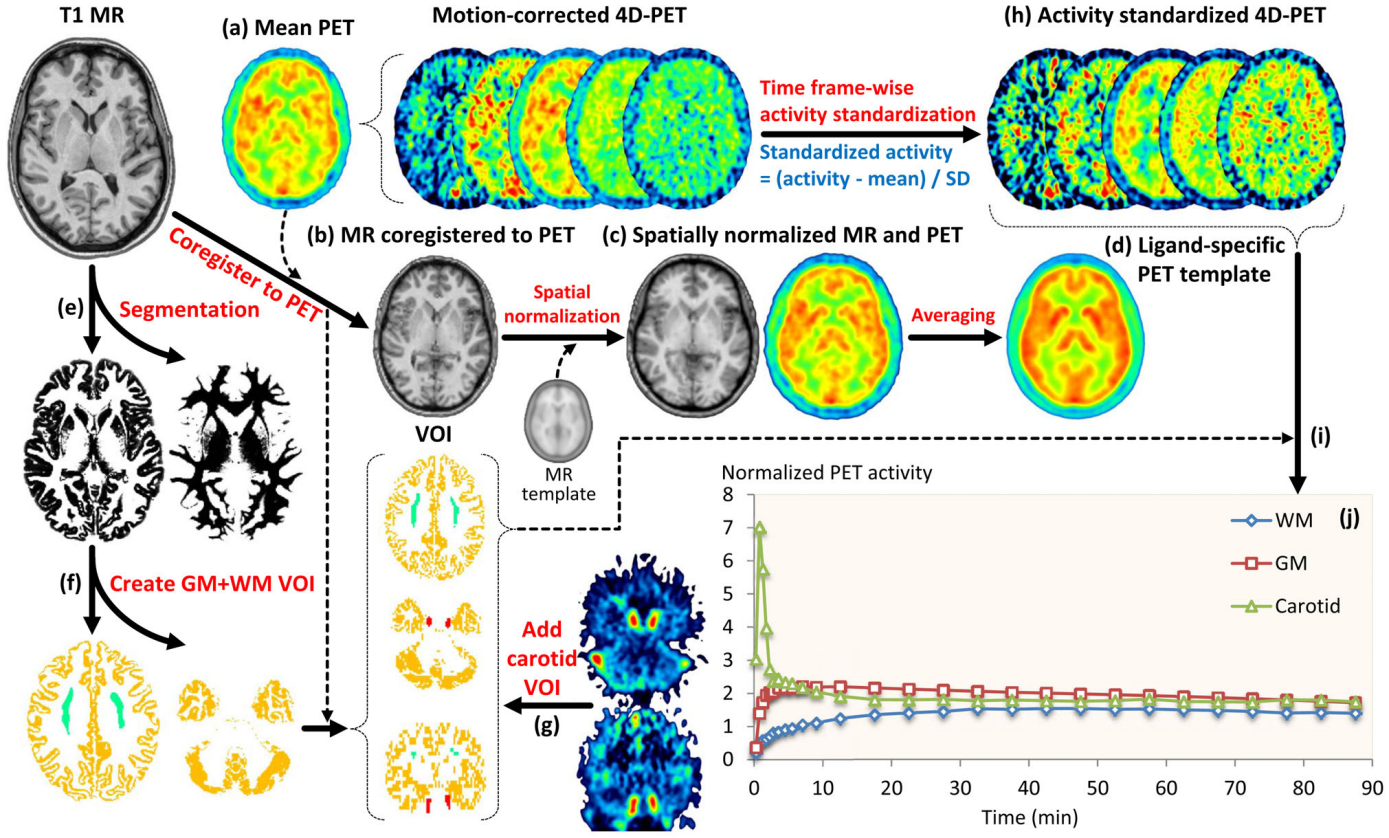
Creation of ligand-specific PET template and time-activity curve (TAC) templates. Mean positron emission tomography (PET) image was created by first averaging the motion-corrected PET dynamic frames (a). The T1-weighted magnetic resonance (MR) image was then coregistered to mean PET image (b). This coregistered mean PET image was spatially normalized (c) based on the transformation parameters from spatial normalization of MR image to the Montreal Neurological Institute (MNI) MRI template. Spatially normalized mean PET images from all 10 subjects were averaged to obtain the final ligand–specific template (d). From automatic MRI segmentation (e), we obtained the volumes of interest (VOIs) for grey and white matter (f). The carotid VOIs were obtained from manual segmentation of the early PET frames (g). Motion-corrected dynamic PET images were standardized by activity (h). The template curves for the three tissues (i) were derived by averaging the TACs of the 10 subjects obtained from each mask applied to the standardized PET images (j).

Dynamic PET images were first realigned to correct for motion. All time frames of the dynamic PET images were then averaged to create a mean PET image (Figure 1a), and the T1-weighted MR image was coregistered to this mean PET image (Figure 1b). The averaged PET images were spatially normalized to the MNI MR template using the transformation parameters of the coregistered MR images (Figure 1c). The ligand-specific spatial template was created by averaging the spatially normalized mean PET images of the 10 subjects (Figure 1d). This ligand specific template for [^11^C](*R*)-rolipram PET was used to obtain the transformation matrix for normalizing each PET image without using the MR image.

#### Creation of the template time-activity curves

We extracted three kinetic tissue models: gray matter, white matter, and blood. The gray and white matter masks were delineated on the individual MR images of the 10 subjects. It should be noted that prior removal of extracerebral tissues, as proposed in an optimized version of this algorithm [19], was not necessary in the present study because blood voxels were readily identified in all scans. The reconstructed T1-weighted MR images were corrected for intensity inhomogeneity and segmented into gray and white matter (Figure 1e). The gray matter mask was created by selecting the voxels showing a probability greater than 0.9. To obtain the white matter mask we first created a map of the amount of spill-over from the gray matter into the white matter. This was done by applying a 3D Gaussian kernel with 7 mm of Full Width at Half Maximum (FWHM) to the gray matter mask image after applying a probability of 0.5 as a threshold. The final white matter mask was created using the voxels within the white matter whose spill-over amount was less than 0.5% of the imaginary uniform activity of gray matter (Figure 1f). To acquire template TACs for blood, we manually drew carotid VOIs on a PET image comprising the averaged first frames (from the second to the fourth) (Figure 1g). The activity of each frame was standardized by subtracting the mean and dividing by the standard deviation of the individual frame (Figure 1 h). Finally, the template curves for the three tissues were derived by averaging the TACs of the 10 subjects obtained from the masks for gray matter, white matter, and carotid applied to the standardized PET image of each subject (Figure 1i and j).

#### Classification of tissue types using supervised clustering

**Figure 2:**
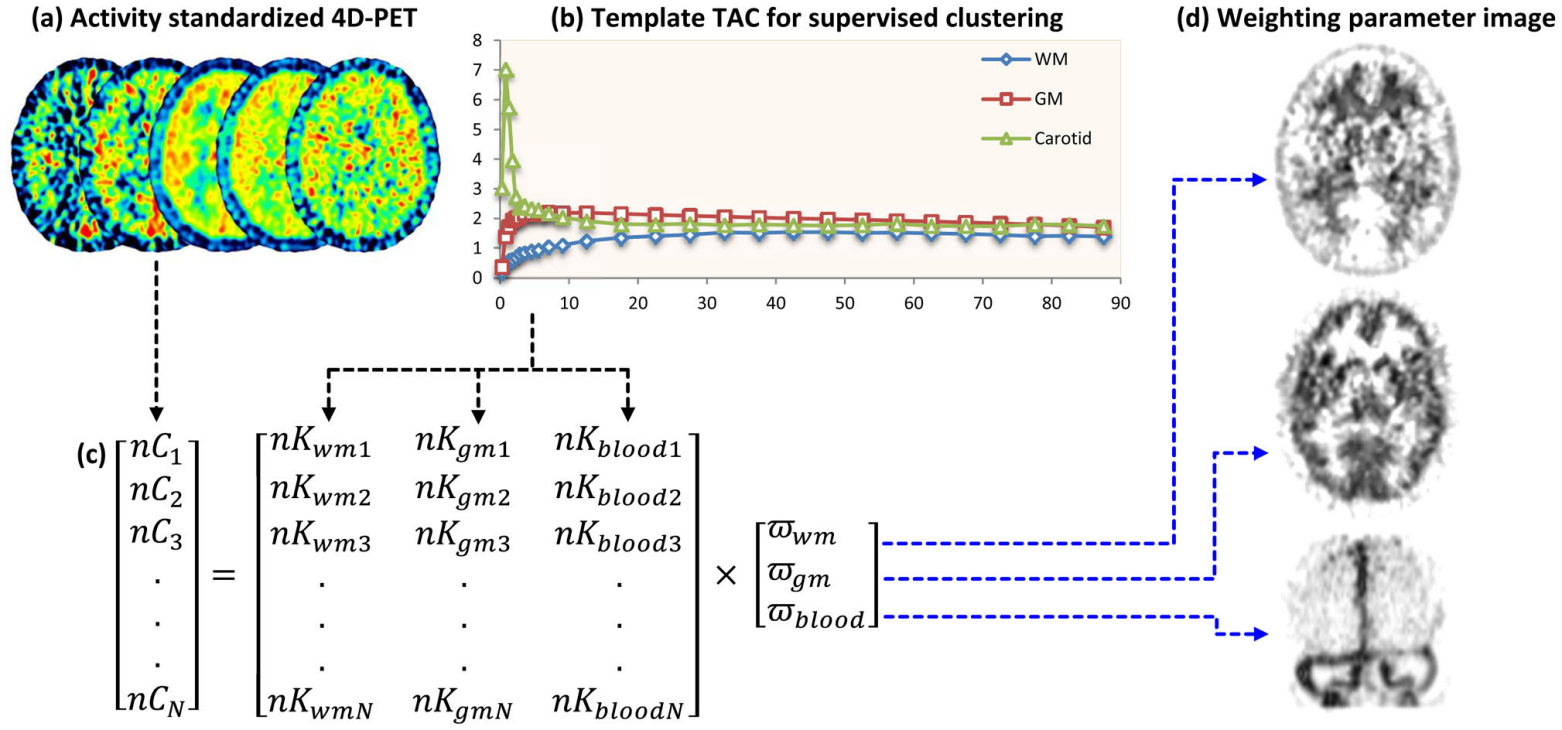
Classification of tissue types using supervised clustering. The time frames of the dynamic positron emission tomography (PET) images were first standardized by activity (a). Then, using the template TACs for the three tissues (b), we calculated the weighting parameters by using a voxel-wise non-negative least squares algorithm (c). A 3D volume with the weighting parameter information for grey matter, white matter, and blood was generated (d).

The supervised clustering procedure was carried out as originally described [12], using in-house software implemented in MATLAB 7.1 (MathWorks, Natick, MA). In brief, all time frames of the dynamic PET images were first standardized by activity (Figure 2a). Then, using the template TACs for the three tissues, weighting parameters were calculated for each tissue using a voxel-wise non-negative least squares algorithm (Figure 2b and c). The weighting parameters describe the similarity to each template TAC in each voxel. Finally, 3D volumes with the information of weighting parameters for gray matter, white matter, and blood were acquired (Figure 2d).

#### Creation of vascular template and template mask for the carotid and surrounding tissue

**Figure 3:**
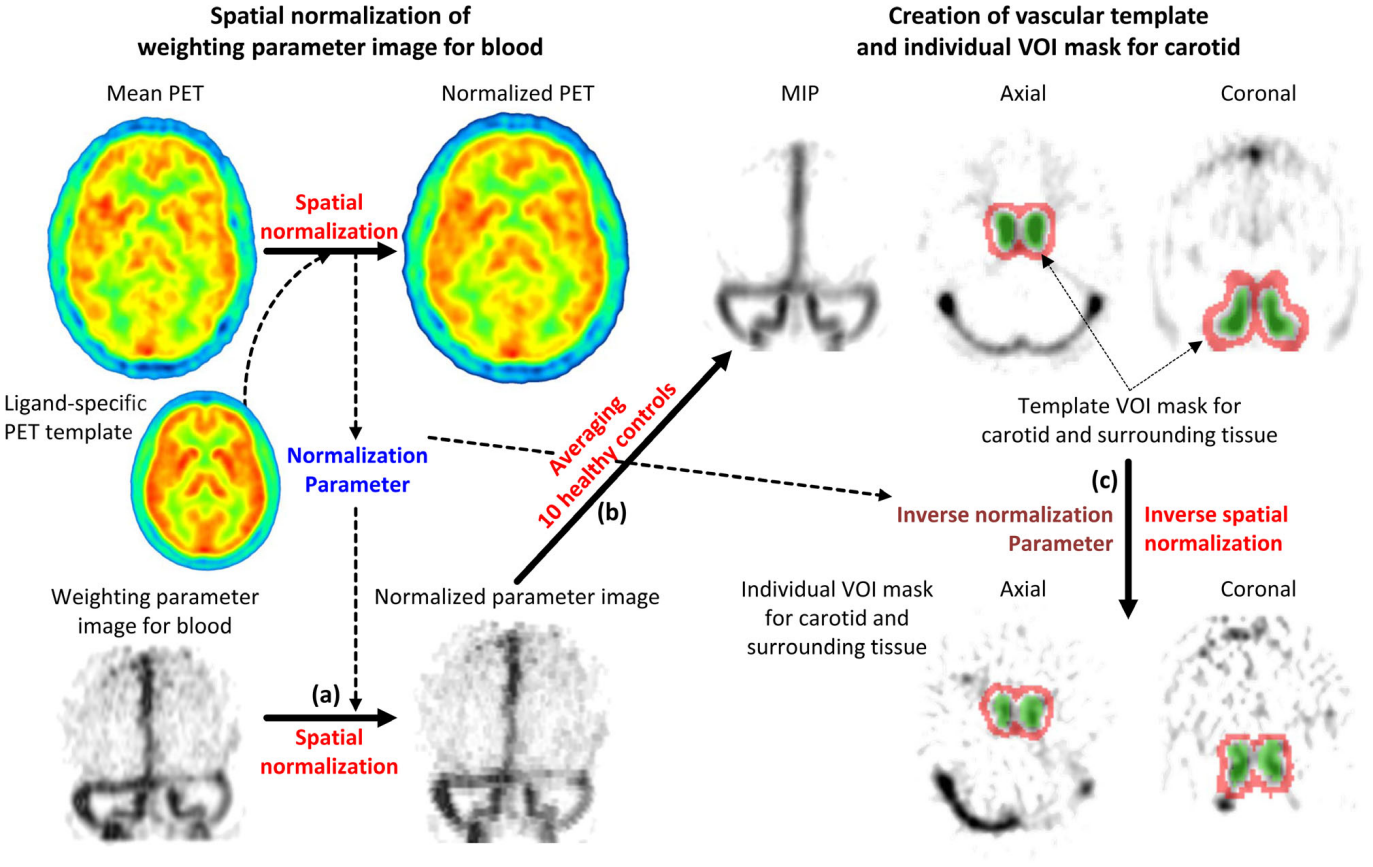
Creation of volumes of interest (VOIs) mask for the carotid artery and its surrounding tissue. The template VOI mask for carotid and surrounding tissue was obtained from the spatially normalized (a) and averaged weighting parameter image for blood (b). Individual VOI masks for carotid and surrounding tissue were created by inverse spatial normalization of the template VOI mask (c). The inverse normalization parameters were derived from the spatial normalization of the average image of dynamic positron emission tomography (PET) to the ligand specific PET template.

The mean PET images were spatially normalized to the ligand-specific PET template and the resulting transformation parameters were used to normalize the images weighted for the blood component (Figure 3a). Spatially normalized blood-weighted images from the 10 subjects were then averaged to create a vascular template image for designing a template mask for the carotid artery and surrounding tissue (Figure 3b).

After manually removing the voxels belonging to the cerebral venous sinuses and voxels considered to be noise from the blood template image, we created a template mask image for the carotid artery by including only the voxels whose parameters were higher than 0.2. For the surrounding tissue mask, a 3D Gaussian kernel with 7 mm of FWHM was first applied to the carotid mask image. The template mask image for the surrounding tissues was then created by selecting the voxels with values between 0.1 and 0.001. The two masks were merged into a single volume to obtain a template VOI mask image for both carotid and surrounding tissue. The inverse spatial normalization parameters derived from the normalization of the individual mean PET image to the ligand-specific template were applied to the template VOI mask. This was done to create individual VOI masks to measure TACs for carotid and surrounding tissue (Figure 3c).

#### 2) Extracting whole blood curve from dynamic PET

Both activity standardization and supervised clustering to extract weighting parameters for blood were conducted for all 51 PET scans from the clinical study. The individual mask images for the carotids and surrounding tissues were created by applying the inverse normalization parameter derived from the spatial normalization of each averaged PET image to the ligand-specific template. Voxels showing more than 50% of weighting parameter values within the carotid mask were selected, and the carotid TAC was obtained by averaging the voxels; these were weighted by their weighting parameter [12]. In addition, within the individual mask of surrounding tissue, the TAC of the surrounding tissue was obtained using the unweighted average of the voxels with less than 0.1% of weighting parameter for blood.

#### 3) Correction for partial volume effect and radiometabolites

The method used here, originally proposed by Chen and colleagues for [^18^F]FDG [20], is very robust with regard to motion artifacts [21], thus making it applicable for imaging patients with dementia or movement disorders. We validated this method for [^11^C](*R*)-rolipram [2], [4], [22]. Briefly, the carotid signal in the images is represented as a linear combination of the radioactivity from the blood and that spilling into the carotid from the surrounding tissue, according to the formula:
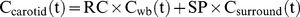
(1)where C_carotid_ and C_surround_ are the radioactivity concentrations in the carotid and the surrounding tissue, respectively; C_wb_ is the radioactivity measured in arterial whole blood; and RC and SP are the recovery and spill-in coefficients, which were estimated with linear least square fitting of C_carotid_, C_wb_, and C_surround_ in equation (1) at the sampling times (t). C_wb_ was measured at 6, 20, 60, and 90 minutes. In one subject, whole-blood IDIF was obtained using only the first three samples (6, 20, and 60 minutes) because the addition of the fourth sample resulted in a curve whose shape was inconsistent with that of an input function. To obtain the concentrations of unchanged parent ligand, a monoexponential function was fitted to the individual parent/whole blood concentration at at 6, 20, 60, and 90 minutes and then multiplied by the whole-blood IDIF.

### Calculation of [^11^C](*R*)-rolipram Binding

Logan analysis was used to calculate *V*_T_ values, which were divided by the individual plasma free fraction (*V*_T_/*f*_P_). Logan-*V*_T_/*f*_P_ values obtained in the control group and MDD patients were compared using repeated measures two-way analysis of variance with regions as the within-subjects (repeating) factor. The analysis was performed using Logan-*V*_T_/*f*_P_ values obtained with reference arterial input function and with IDIF. Statistical analyses were performed using IBM SPSS Statistics 20 (Armonk, NY).

## Results

**Figure 4:**
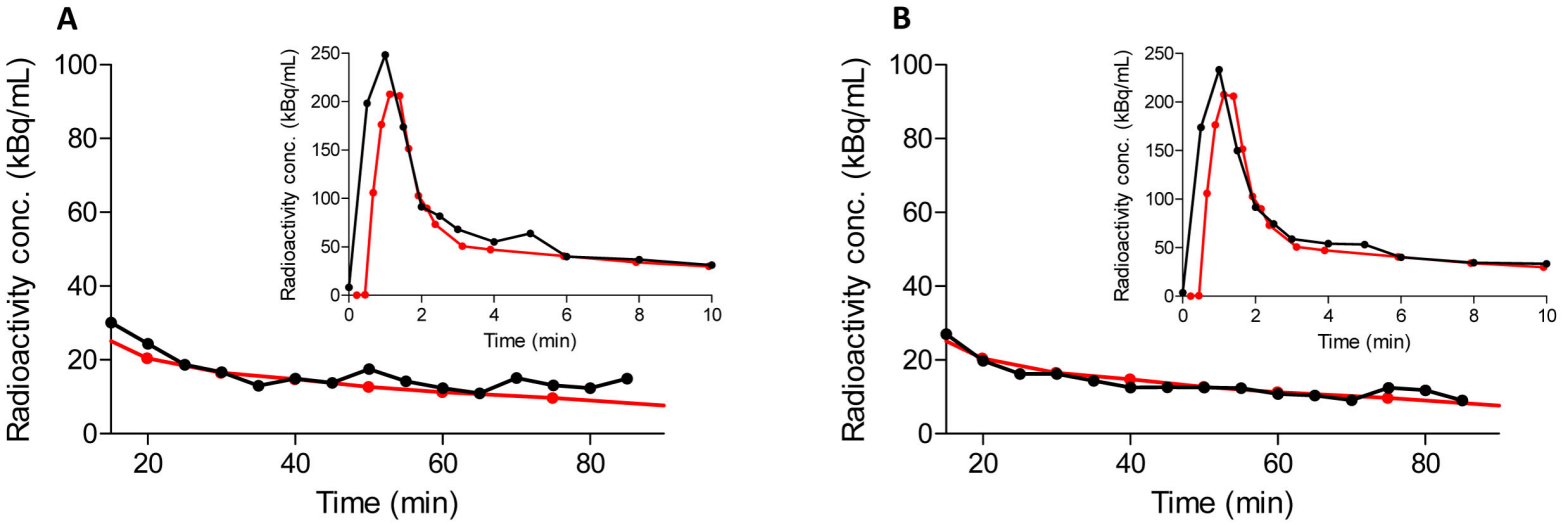
Manual and cluster-IDIF in a representative subject. Metabolite corrected arterial input function (in red) and image-derived input function (IDIF) (in black) in a representative subject. The insets show the curves from 10 to 90 minutes. Main graphs show the early part of the curves until 10 minutes. Carotid and background segmentation was performed manually (A) or with the clustering algorithm (B). In most subjects, the automatic IDIFs were slightly less noisy.

The clustering algorithm clearly segmented the carotid arteries in all PET scans. Although the height of the IDIF peaks generally presented some variations compared to the arterial peak, at later times the image-derived curves followed the full arterial curves well. Both visual inspection as well as comparison of IDIFs obtained from manual drawings [4] found that IDIFs obtained with the clustering procedure were less noisy for most subjects (Figure 4). To quantitatively support our visual impression, we calculated the sum of the squared distance at each time point after 10 minutes between the reference arterial curve, manual-IDIF, and cluster-IDIF. The mean sum of the squared distance expressed in kBq/mL from the 51 subjects for cluster-IDIF (160.3±142.4) was lower than that for manual-IDIF (172.6±138.1). The cluster-IDIF gave a lower value in 34 of 51 subjects; the likelihood of this occurring by chance is approximately 1%.

**Figure 5:**
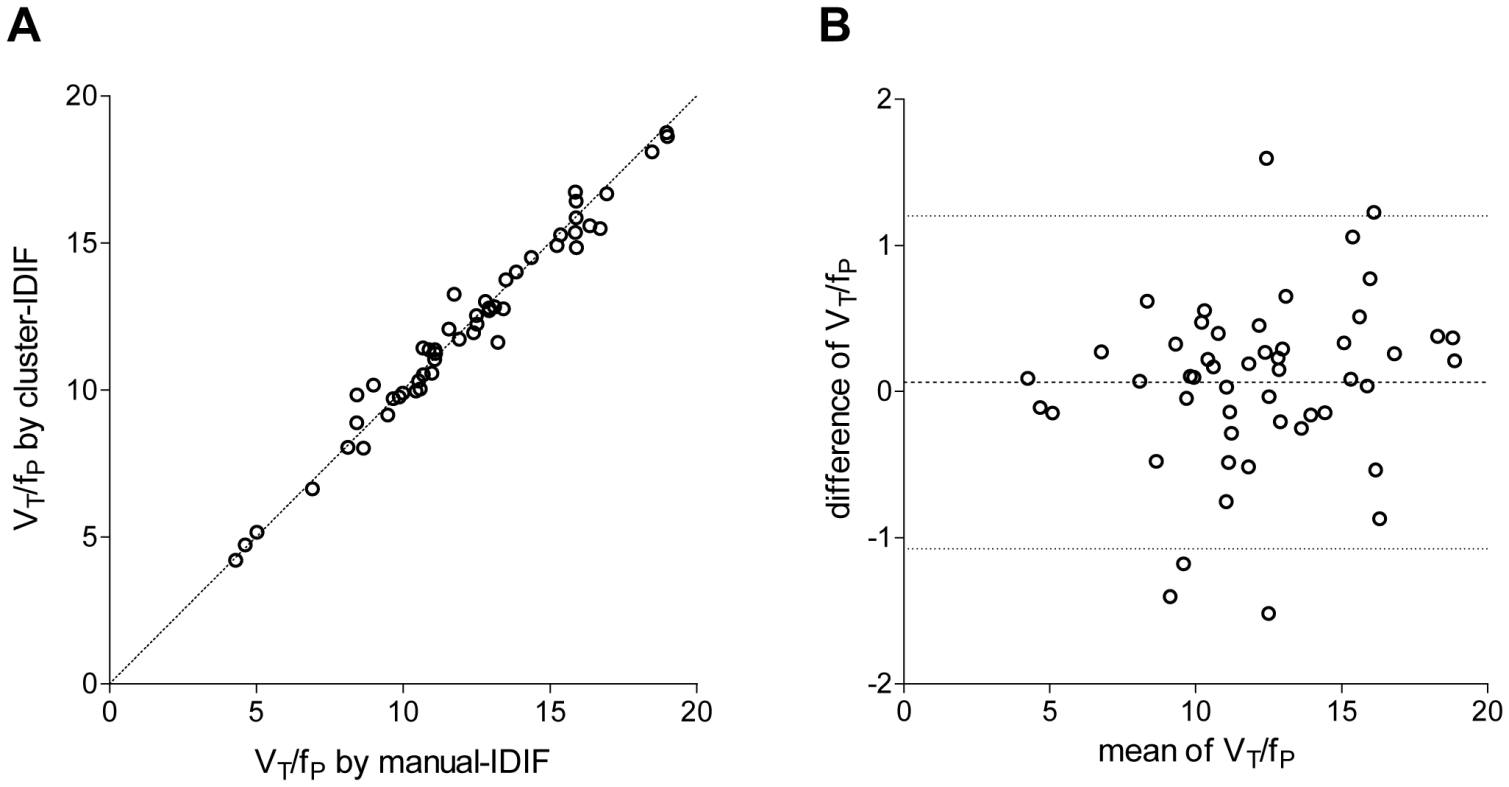
Correlation and agreement between the Logan-*V*_T_/*f*_P_ values derived from arterial input function and cluster-IDIF. (A) The image-derived input functions (IDIFs) derived from cluster segmentation yielded Logan-*V*_T_/*f*_P_ values that were well-correlated with those derived from the reference arterial input function (Pearson’s correlation, p<0.0001). Each of the 51 points represents an average *V*_T_/*f*_P_ value of grey matter in a single subject. (B) The Bland-Altman plot shows that the two measurements are in good agreement, with no proportional bias through the range of measurements. The dotted line in the middle shows the mean difference; the other two dotted lines show the 95% confidence interval.

The estimated Logan-*V*_T_/*f*_P_ values obtained via cluster-IDIF were very similar to the reference values (Pearson’s correlation, p<0.0001, R^2^ = 0.976 in Figure 5A) and showed no proportional bias through the range of measurements (Figure 5B). The estimated/reference Logan-*V*_T_/*f*_P_ ratio was 0.99±0.04, and these results were equally accurate for healthy controls (0.98±0.04) and MDD patients (1.01±0.04). Thirty-nine of 51 subjects had a *V*_T_/*f*_P_ error of <5%, 11 had an error between 5 and 10%, and one had an error of 11%.

Logan-*V*_T_/*f*_P_ values obtained with cluster-IDIF showed a widespread decrease of about 20% [^11^C](*R*)-rolipram binding in the MDD group (mean *V*_T_/*f*_P_ in controls: 13.2±3.10 mL/g, MDD: 11.0±3.30, two-way ANOVA p = 0.017 for group difference in the 10 brain regions); these findings echoed previous results obtained using the full input (controls: 13.5±3.10 mL/g, MDD: 10.9±3.21; p = 0.005) as well as IDIF by manual segmentation (controls: 13.4±3.20 mL/g, MDD: 10.9±3.33; p = 0.008) [4].

## Discussion

This study found that an SVCA-derived cluster-IDIF provided good carotid segmentation for [^11^C](*R*)-rolipram brain PET scans. After partial volume and radiometabolite correction, the carotid TACs were used as an IDIF to estimate [^11^C](*R*)-rolipram binding. The technique was validated on a large number of subjects. Furthermore, we were able to replicate the results of a previously published clinical protocol comparing healthy subjects and MDD patients [1], which showed a global reduction of [^11^C](*R*)-rolipram binding (of about 20%) in MDD patients compared to controls.

The main advantage of automatic segmentation is that it completely removes intra- and inter-operator variability. Although this variability is quite small (a few percentage points at most) when using Chen’s method [20], results obtained with manual segmentation are nevertheless always influenced by the shape of the VOIs and the number of slices included because the carotid TACs are ultimately fitted to blood samples.

In most subjects in this study, the IDIF obtained with the clustering algorithm was also of better quality (less noisy) than the IDIF drawn manually on the same scans [4] (Figure 4), as demonstrated by a lower sum of the squared distance between the reference arterial curve and both the manual- and cluster-IDIFs. Indeed, carotid VOIs obtained from automatic segmentation would likely include a higher number of vascular voxels and a lower number of background voxels than when segmentation is obtained manually. This improved IDIF quality, however, did not translate into a more accurate estimate of the final Logan-*V*_T_ results. In fact, the differences in the area under the curve were minimal; regardless, all input functions were fitted with a tri-exponential function before modeling, thus effectively eliminating noise. It can also be argued that if a given IDIF technique yields significantly different results on the basis of a difference of a few voxels between manual or automatic segmentation, then it would be too unstable to be used. Our results nevertheless suggest that voxel selection with the clustering algorithm is not only more reproducible, but also more accurate than manual segmentation. These results further echo those obtained by Chen and colleagues using an automatic carotid segmentation with independent component analysis (ICA) [23]. The use of ICA resulted in a faster and more objective carotid segmentation, although the results were not significantly better than those obtained by simple manual segmentation [20].

Another advantage of automatic segmentation is the amount of time saved. When the procedure is entirely automated, a simple computer script allows a whole population of subjects to be analyzed at once, as was done in the present study.

Notably, the present technique does not require coregistered anatomical images. MRI and computer assisted tomography (CT) scans have sometimes been used to help delineate the carotid arteries [24], [25]. However, the carotid is a very small and elastic structure that may present significant coregistration challenges [26], [27]; direct segmentation from PET images is therefore preferable. New hybrid PET/MRI machines would allow the position and size of the carotid vessels to be determined more accurately, and some authors have argued that this might facilitate IDIF estimates, possibly in a completely non-invasive way [28], [29]. In our opinion, however, a more accurate coregistration with MRI will not improve IDIF estimates because of the intrinsic limits of PET technology. For instance, even a theoretically perfect spatial delineation of the carotids would not solve the problem of poor temporal resolution and the separation of the parent concentration in plasma from total blood radioactivity. Poor temporal resolution would not allow a good estimate of the rapidly changing early part of the input function (the peak). For radioligands with a fast washout from the vascular compartment, an accurate estimation of the peak is necessary when using a Logan analysis because a large part of the area under the curve is found under the peak [26]. Moreover, even if a perfect partial volume effect recovery could be obtained from the images, the reference recovery coefficient would already be available with a simple measurement of the whole-blood concentration in the blood samples that must, regardless, be collected for radiometabolite correction.

It should be noted that good segmentation can likely be obtained with other radioligands, PET scanners, and subject populations. Indeed, the extant literature shows that the supervised clustering method has been acquired with different scanners, as well as successfully applied to populations with brain atrophy [30]–[32]. However, after carotid segmentation, partial volume and spill-in correction must be performed. For [^11^C](*R*)-rolipram, excellent results were obtained using the method developed by Chen and colleagues [20], using both a high resolution research tomograph [2] and a machine with a lower resolution such as the one used in the present study. However, partial volume correction methods are very tracer-specific and an appropriate technique must be found for each tracer [26]. To correct for partial volume effect, some blood samples are necessary to estimate an IDIF, given that non-invasive methods are generally less reliable [22], [33]. In order to avoid an arterial line, the best option would be to use venous, instead of arterial, samples. However, arterovenous equilibrium is uncommon [34]. In particular, [^11^C](*R*)-rolipram does not demonstrate satisfactory equilibrium; thus, some arterial samples are still necessary to correct for partial volume effect [4].

In summary, cluster-IDIF is capable of estimating Logan-*V*_T_/*f*_P_ in [^11^C](*R*)-rolipram PET clinical scans as well as full arterial input function. Nevertheless, the applicability of this technique must be tested for each new radioligand. We believe that good segmentation of blood voxels will not be difficult to achieve for most radiotracers. However, the main obstacle to widespread application of this technique is finding a reliable partial volume effect correction of the raw carotid curves.
